# Models of Regional Habitat Quality and Connectivity for Pumas (*Puma concolor*) in the Southwestern United States

**DOI:** 10.1371/journal.pone.0081898

**Published:** 2013-12-18

**Authors:** Brett G. Dickson, Gary W. Roemer, Brad H. McRae, Jill M. Rundall

**Affiliations:** 1 Conservation Science Partners, Inc., Truckee, California, United States of America; 2 Lab of Landscape Ecology and Conservation Biology, School of Earth Sciences and Environmental Sustainability, Northern Arizona University, Flagstaff, Arizona, United States of America; 3 Department of Fish, Wildlife and Conservation Ecology, New Mexico State University, Las Cruces, New Mexico, United States of America; 4 The Nature Conservancy, North America Region, Seattle, Washington, United States of America; Bangor University, United Kingdom

## Abstract

The impact of landscape changes on the quality and connectivity of habitats for multiple wildlife species is of global conservation concern. In the southwestern United States, pumas (*Puma concolor*) are a well distributed and wide-ranging large carnivore that are sensitive to loss of habitat and to the disruption of pathways that connect their populations. We used an expert-based approach to define and derive variables hypothesized to influence the quality, location, and permeability of habitat for pumas within an area encompassing the entire states of Arizona and New Mexico. Survey results indicated that the presence of woodland and forest cover types, rugged terrain, and canyon bottom and ridgeline topography were expected to be important predictors of both high quality habitat and heightened permeability. As road density, distance to water, or human population density increased, the quality and permeability of habitats were predicted to decline. Using these results, we identified 67 high quality patches across the study area, and applied concepts from electronic circuit theory to estimate regional patterns of connectivity among these patches. Maps of current flow among individual pairs of patches highlighted possible pinch points along two major interstate highways. Current flow summed across all pairs of patches highlighted areas important for keeping the entire network connected, regardless of patch size. Cumulative current flow was highest in Arizona north of the Colorado River and around Grand Canyon National Park, and in the Sky Islands region owing to the many small habitat patches present. Our outputs present a first approximation of habitat quality and connectivity for dispersing pumas in the southwestern United States. Map results can be used to help target finer-scaled analyses in support of planning efforts concerned with the maintenance of puma metapopulation structure, as well as the protection of landscape features that facilitate the dispersal process.

## Introduction

Loss of habitat is one of the leading causes of species endangerment, and disruptions in connectivity are expected to increasingly heighten this risk as the Earth warms, necessitating novel conservation strategies [1], [2]. Maintaining connected habitats by conserving and restoring linkage zones or corridors is becoming one of the most common strategies for mitigating human-caused landscape and climate change [3]. Regional conservation planning efforts are thus needed to identify where vital linkages are at risk of being severed or disrupted over extensive habitat networks.

Mammalian carnivores are important as keystone species in many ecosystems [4], but also are threatened by adverse changes in habitat quality and connectivity globally [5]. Furthermore, because the conservation of spatially extensive, heterogeneous landscapes needed to support viable populations of large carnivores would protect other species whose habitat requirements are met within a much smaller spatial extent [6], conserving carnivores can be an effective strategy for protecting habitat necessary for other species [7], [8]. Methods and tools that can predict the distribution and quality of both high-quality habitat and its connectedness are thus needed not only for carnivore conservation, but for broader conservation objectives as well.

In Arizona and New Mexico, USA, puma (*Puma concolor*) populations are well distributed and occupy most of the contiguous habitats available to them or their prey [9]. In the southern portions of these states, habitats able to support pumas are found in small to medium-sized mountain ranges (i.e., ‘Sky Islands’) embedded in a landscape matrix of desert that pumas do not regularly occupy. Varying levels of dispersal may connect these subpopulations, with the quality of movement pathways linking them being influenced by a diverse set of natural and artificial landscape features [10]. Like other large carnivores, pumas are sensitive to loss of habitat and disruption of the pathways that connect their populations [11]–[13]. Thus, the viability of puma populations inhabiting these isolated mountain ranges is very likely dependent upon maintaining connections among them [9], [14].

We present an approach for modeling of habitat quality and connectivity for pumas in Arizona and New Mexico. We chose to model pumas because they are a good surrogate for species occupying the more rugged and wooded vegetation communities in the southwestern United States. Pumas need large areas to maintain viable populations and they use a broad range of habitats when dispersing out of their natal range [10]. Across the southwestern U.S., these habitats are becoming further fragmented or isolated by urbanization and other forms of human development [15], such as transportation corridors [16], [17], border security infrastructure [18], and energy development [19]. Puma populations are of concern to many conservation and resource management stakeholders interested in conserving wide-ranging species and the remaining landscapes they inhabit. In Arizona and New Mexico, pumas are managed by state wildlife agencies as a game animal, and their population dynamics are adversely impacted by sport harvest [20], [21].

Our objectives were to 1) collaborate with regional experts to determine the variables most likely to influence puma habitat quality, location, and permeability to movement; 2) use this information to develop spatially explicit models and maps of habitat quality and patch location; and 3) extend current connectivity modeling approaches, based on circuit theory, to identify areas important for maintaining connectivity both for direct dispersal between neighboring pairs of habitat patches and for keeping the entire network of habitat patches connected. Importantly, we aimed to produce models that could be used as *a priori* hypotheses about regional connectivity for pumas and tested with independent data sets or applied to other regions and taxa.

## Materials and Methods

### Study Area

**Figure 1:**
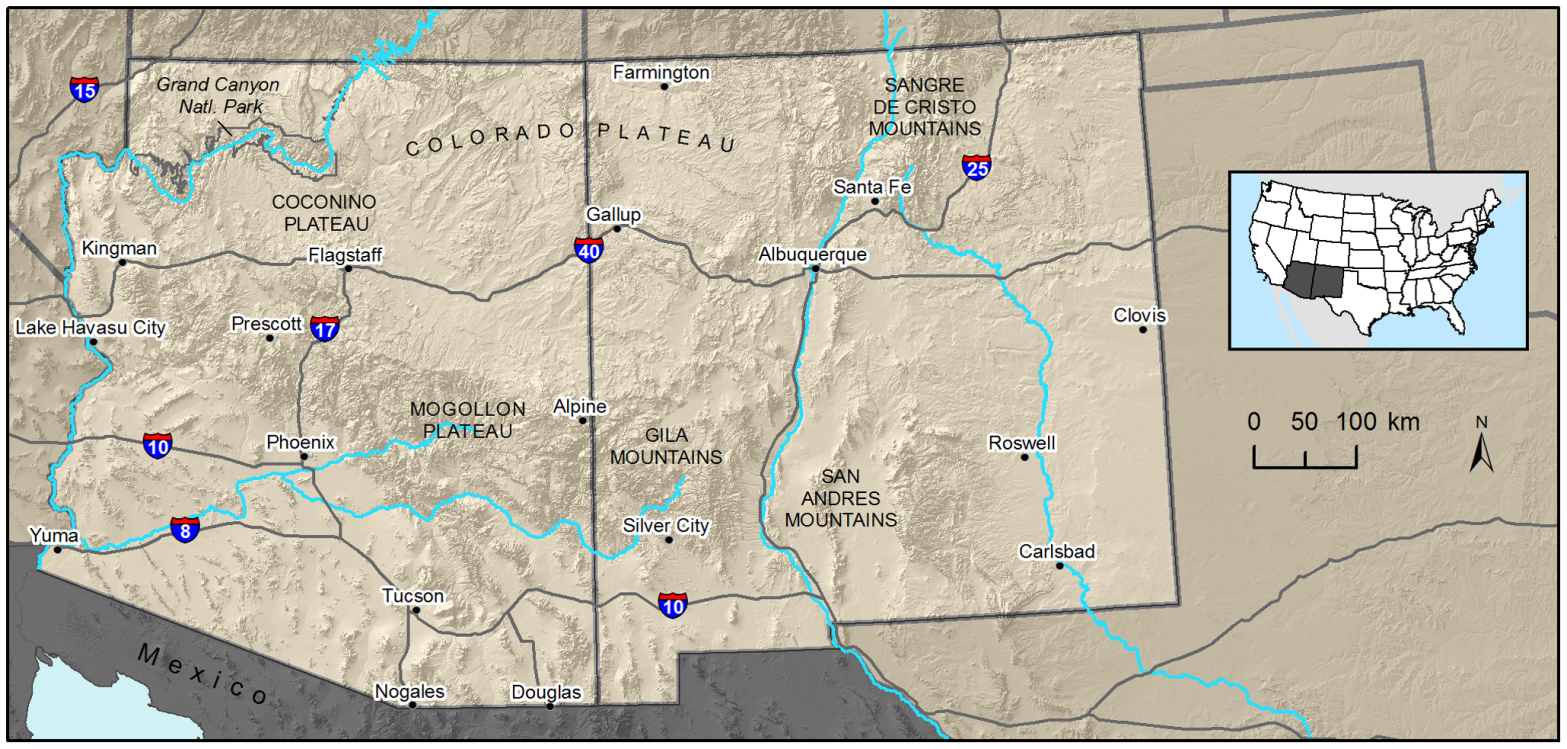
Map of study area used to estimate regional connectivity for pumas. The study area encompassed all of Arizona and New Mexico, USA, or approximately 611,300^2^. Background maps were produced using data obtained from the U.S. Geological Survey’s Earth Resources Observation and Science Center.

We modeled puma habitat and connectivity across Arizona and New Mexico, an area of approximately 611,300 km^2^ (Fig. 1). This area encompasses prominent landscape features such as the Grand Canyon, the Coconino and Mogollon Plateaus, the Sangre de Cristo Mountains, several other large, typically forested mountain ranges, the Rio Grande River Valley and a diversity of lowland vegetation types capable of supporting the movement and space-use requisites of pumas and their primary prey, mule deer (*Odocoileus hemionus*; [9]). Topography in these mountain ranges is rugged and elevations range from 25 m in the southwestern corner of Arizona to 4,012 m at the top of Wheeler Peak in northern New Mexico (mean elevation = 1,863, ±1SD = 1,084). Over 50% of the study area is in public ownership, including iconic national parks like Grand Canyon National Park, large military installations, other federally owned lands, lands owned by Native American tribes and the states of Arizona and New Mexico, interspersed with private lands. As of 2011, three incorporated cities (Phoenix, Tucson, and Albuquerque) had human populations between 500,000–1,500,000.

### Integrating Expert Knowledge

Existing empirical data on puma habitat use and dispersal were insufficient to estimate habitat quality and connectivity at the scale of our analysis or improve upon a comprehensive base of regional knowledge [22]. We thus developed models of habitat quality and connectivity that incorporated knowledge from five individuals with considerable expertise on the ecology of pumas in the study region (Text S1). We elicited and integrated their knowledge using a multi-criteria framework and an iterative three-step process of variable specification, model building, and model review (see [23], [24]; Text S1, Figure S1). In the first step, each expert was tasked with specifying up to four unique habitat variables that they believed were most likely to influence habitat quality and permeability (or conductance, as derived below) for pumas in the Southwest. Often, multiple experts selected similar (potentially correlated) variables and so we synthesized this information with their review and approval [23]. This first step resulted in the description of seven habitat variables that fell into three broad categories: land cover, terrain, and anthropogenic features. In the second step, described in detail below, we asked each expert to provide a score for each class within a given habitat variable (e.g., ‘forest’ land cover type, ‘ridgeline’ topographic position), and also assign a weight to each of the seven variables. As a third step, each expert was encouraged to modify or refine preliminary or final model outputs, if necessary.

### Habitat Variables

We used expert knowledge and a GIS (ArcGIS® v10.0, Esri, Redlands, CA, USA) to determine and derive seven habitat variables from multiple data layers available for our study area, including a 10-km buffer to accommodate analytical edge effects. Each of the data layers was resampled from its native resolution (typically, 30 m) or format (e.g., vector) to a resolution of 900-m grid cell size prior to analyses using moving window functions in the GIS (e.g., to calculate the average value for a given cell within a 30×30 m-cell neighborhood). The seven habitat variables were divided into three broad categories: land cover, terrain and anthropogenic features. Many of these or similar variables have previously been reported in the literature as important factors influencing puma habitat quality and movement.

#### Land cover

We used spatial data on existing ecological systems derived by the Interagency Landfire Project (www.landfire.gov; [25]) to characterize 12 dominant land cover types (new habitat variable = COVTYPE; Table 1). These 12 land cover types included all of the dominant land cover types previously used to elucidate patterns of movement and vegetation selection by pumas, for example, in southern California [16], [26] and Arizona [17].

**Table 1:** Expert-defined habitat variables (data layer name) and associated classes, and average (±1SD) habitat quality and permeability scores used to estimate connectivity for pumas in Arizona and New Mexico.

Habitat variable	Quality	Permeability
*Land cover (COVTYPE)*		
Open water	0.0 (0.0)	25.0 (28.9)
Developed	70.0 (49.7)	113.8 (66.5)
Barren	61.3 (109.3)	208.8 (139.8)
Agriculture	268.8 (128.1)	418.8 (140.5)
Woodland	912.5 (103.1)	962.5 (47.9)
Forest	851.3 (170.8)	951.3 (54.8)
Shrub	700.0 (70.7)	848.8 (160.7)
Desert scrub	577.5 (349.8)	752.5 (222.9)
Chaparral	671.3 (266.4)	746.3 (270.0)
Shrub-steppe	450.0 (229.1)	883.3 (125.8)
Grassland	286.3 (146.4)	607.5 (253.4)
Riparian	670.0 (263.2)	917.5 (165.0)
*Terrain ruggedness (degrees; RUGGED)*		
0.00–0.47	100.0 (0.0)	100.0 (0.0)
0.48–0.93	200.0 (0.0)	200.0 (0.0)
0.94–1.63	300.0 (0.0)	300.0 (0.0)
1.64–2.57	400.0 (0.0)	400.0 (0.0)
2.58–3.62	500.0 (0.0)	500.0 (0.0)
3.63–4.78	600.0 (0.0)	600.0 (0.0)
4.79–5.95	700.0 (0.0)	700.0 (0.0)
5.96–7.00	750.0 (57.7)	775.0 (50.0)
7.01–8.17	775.0 (150.0)	850.0 (100.0)
8.18–29.76	800.0 (245.0)	850.0 (238.0)
*Large barriers (BARRIERS)*		
Major highways	2.5 (5.0)	62.5 (92.4)
Large canals and ditches	80.0 (98.0)	148.8 (84.7)
Lakes and reservoirs	0.0 (0.0)	17.5 (22.2)
*Topographic position index (TPI)*		
Canyon bottom	855.0 (290.0)	955.0 (90.0)
Gentle slope	586.3 (118.0)	801.3 (234.9)
Steep slope	732.5 (92.5)	726.3 (337.3)
Ridgeline	755.0 (126.9)	902.5 (81.8)
*Road density (km/km^2^; ROADS)*		
0.00–0.30	1000.0 (0.0)	1000.0 (0.0)
0.31–0.45	900.0 (0.0)	925.0 (50.0)
0.46–0.57	800.0 (0.0)	850.0 (100.0)
0.58–0.68	700.0 (0.0)	775.0 (150.0)
0.69–0.79	600.0 (0.0)	600.0 (0.0)
0.80–0.90	500.0 (0.0)	500.0 (0.0)
0.91–1.10	400.0 (0.0)	400.0 (0.0)
1.20–1.30	300.0 (0.0)	300.0 (0.0)
1.40–1.70	200.0 (0.0)	200.0 (0.0)
1.80–9.60	100.0 (0.0)	100.0 (0.0)
*Distance to water (meters; WATER)*		
0.0–2950	875.0 (250.0)	700.0 (355.9)
2950–5150	825.0 (221.7)	650.0 (300.0)
5160–7360	775.0 (206.2)	600.0 (244.9)
7370–9930	725.0 (206.2)	550.0 (191.5)
9940–12900	675.0 (221.7)	500.0 (141.4)
13000–16200	500.0 (0.0)	450.0 (100.0)
16300–20200	400.0 (0.0)	375.0 (50.0)
20300–25700	300.0 (0.0)	300.0 (0.0)
25800–34900	200.0 (0.0)	200.0 (0.0)
35000–93800	100.0 (0.0)	100.0 (0.0)
*Human population density (individs/block; HUMANS)*		
0.0	1000.0 (0.0)	1000.0 (0.0)
1–614	900.0 (0.0)	925.0 (50.0)
615–1230	825.0 (50.0)	850.0 (100.0)
1240–1840	725.0 (50.0)	725.0 (50.0)
1850–2460	575.0 (50.0)	625.0 (50.0)
2470–3070	525.0 (50.0)	525.0 (50.0)
3080–3680	475.0 (150.0)	425.0 (50.0)
3690–4300	275.0 (50.0)	300.0 (0.0)
4310–6140	175.0 (50.0)	200.0 (0.0)
6150–156556	75.0 (50.0)	75.0 (50.0)

#### Terrain

Throughout their range, pumas use a complex mosaic of terrain features to facilitate and conceal their predatory and movement behaviors (see [9]). We used U.S. Geological Survey National Elevation Data (NED) and the Spatial Analyst extension to ArcGIS® to measure elevation, slope (in degrees), and terrain ruggedness (in degrees). We computed terrain ruggedness (RUGGED) as the standard deviation of slope within each 900-m cell. After Dickson and Beier [27], we also used elevation and slope estimates from the NED to derive a Topographic Position Index (TPI) that discriminated between four major classes of terrain: canyon bottom, gentle slope, steep slope, and ridgeline.

#### Anthropogenic features

Numerous road types, including freeways, have been found to influence the movement [16], [28] and genetic structure of pumas and other large, vagile carnivores [29]. Proximity to perennial water sources and areas densely populated by humans also are known affect the movement behavior of pumas [26], [28], [30]. We estimated the density (km/km^2^, 10-km radius) of all road types (ROADS) using U.S. Census Bureau TIGER/Line files® (2012; http://www.census.gov/geo/maps-data/data/tiger-line.html) and a simple density calculation function in ArcGIS. We identified major highways, including freeways and other high-speed (posted speed limit ≥88.5 km/hr) paved roads with ≥4 lanes, large canals or ditches, and large lakes or reservoirs using both the National Transportation Atlas Database (2011; http:www.bts.gov/programs/geographic_information_services) and the National Hydrography Dataset (NHD, 2008; http://nhd.usgs.gov), and added these features to the BARRIERS habitat variable. Therefore, we used the NHD to compute the distance (in meters) to all major perennial water features (e.g., lakes, rivers, springs) on the study area (WATER). We included WATER in the anthropogenic feature classification because many of the water features on the study area have been modified or impacted by humans. Human population density (HUMANS, individuals/block) was estimated from data available from the U.S. Census Bureau (2010; http://www.census.gov/geo/maps-data/data/tiger.html).

### Quantifying Habitat Quality and Large Habitat Patches

We developed an expert-based model of habitat quality for pumas using the seven habitat variables described above. On a continuous scale of 0–1000, each expert scored the relative likelihood that each class of habitat variable could support or sustain the day-to-day behaviors of an individual puma with an established home range (Table S1). Scores of 1000 indicated ‘most likely’ and scores of 0 indicated ‘not capable’. As an initial guide, we used a quantile classification to divide the distribution of cell values for the variables (i.e., layers) RUGGED, ROADS, WATER, and HUMANS into 10 classes of quality ranging from a score = 100 (lowest) to 1000 (highest).

We used a modification to the GIS-based Weighted Linear Combination (WLC) procedure [31] to first average the scores assigned by experts to each habitat class and to weight and combine habitat variables into a final data layer that depicted habitat quality. Next, individual experts assigned a relative rank or ‘importance value’ (on a continuous scale of 0 to 1000) to each of the seven habitat variables (Table S2). We then computed a ‘swing weight’ (*sensu* [31]) for each variable by dividing its importance value by the sum of all importance values. This weighting approach directly incorporates trade-offs among variables and the differences in their value ranges [31]. Swing weights are derived by asking an expert to compare a relative change, or swing, from the lowest to highest quality class of a given habitat variable to a similar change in quality between classes within another habitat variable, and score the importance of all variables accordingly. We next created a preliminary habitat quality layer by calculating the average importance value from among all experts, computing a new swing weight for each layer, and then multiplying this value by the average, expert-defined habitat quality score at each cell. We then added the products for each of the final layers together. Finally, we reclassified these new values using four quartile breaks in the data distribution, where the 75^th^ percentile represented the highest quality habitat. We used this more parsimonious classification (1 = low and 4 = high) to generate our final habitat quality model.

To characterize large, high-quality habitat patches capable of supporting the minimum prey and cover requirements for dispersing pumas, we first used a circular moving window and focal sum operation in the GIS to identify contiguous areas of the highest quality habitat that were within a 5-km radius of each 900-m cell on the study area. We used this distance because it was similar to the radius of the average circular home range derived from home range estimates for 30 female pumas in the San Andres Mountains of southern New Mexico [9]. The resulting map of high quality habitat patches was then provided to each expert for review and modification if they thought necessary.

### Quantifying Habitat Permeability and Connectivity

We estimated how permeable different landscape features were to puma movement by first asking experts to score the relative likelihood of each class of habitat variable to permit the movement of an individual dispersing puma through the dominant characteristics of that class (see Text S1, Table S1). We again used a quantile classification method to initially divide the distribution of cell values for the RUGGED, ROADS, WATER, and HUMANS layers into 10 quality classes varying from 100 to 1000. We then assigned expert-defined importance values and calculated weights for each of the final seven habitat variables using the WLC procedure (Table S2). We derived our final permeability layer by computing the average swing weight among all experts and multiplying this value by the average expert-defined permeability at each cell and then added the final layers together. Prior to implementing a connectivity model, we allowed the average, expert-defined quality score for ‘developed’ areas in the COVTYPE layer to supersede (i.e., ‘trump’) all other values in the final permeability layer. Similarly, we merged the scores for the BARRIERS layer with the final permeability layer and allowed these values to supersede all underlying values, including developed areas.

To estimate connectivity for pumas, we applied concepts from electronic circuit theory using *Circuitscape* software (v3.5, www.circuitscape.org). The software reads a raster map of conductance (reflecting how permeable each cell is to movement) and replaces cells connected by dispersal with nodes connected by resistors [32]. When current is injected into a source patch and allowed to flow to a destination patch, current values at each intervening cell can be interpreted in terms of the probability that a random walker would pass through the cell if it started at the source and moved until it reached the destination [32]. Higher densities of current between habitat patches indicate areas through which successful dispersers are more likely to move. Greater connectivity among populations or habitat patches is predicted when a larger number of connected pathways are available. Locations where current densities are high indicate ‘pinch points’, i.e. areas that act as bottlenecks to movement or where alternative pathways are not available. Pinch-points can be the result of both natural and human-made landscape features, and may represent conservation priorities because their loss can disproportionately disrupt connectivity.

Our application of circuit theory used current flow among high-quality habitat patches to identify important areas for maintaining connectivity both for movements among habitat patch pairs and for maintaining connectivity across the entire network of habitat patches in the study area. To do this we separately mapped current flow between neighboring habitat patches and among all patch pairs in the network. In each case we allowed 1 Amp of current to flow between each patch pair, using our expert-defined permeability layer as the conductance raster [32]. For neighboring patch pairs, we mosaicked current flow into a single map showing maximum flow between any patch pair; current flow between core areas in close proximity to one another dominates in this case. Modeling current flow using this method of maximum pairs is useful for identifying movement ‘pinch points’ between specific patch pairs, irrespective of their importance in the larger network of patches [32]. For analyses across the entire habitat network, we added current flow between all pairs of habitat patches to produce a map of cumulative current flow among all possible pairs. Modeling cumulative current flow provides a quantitative means of evaluating the importance of both patches and intervening areas in the matrix for maintaining connectivity of the entire network of habitat patches in the study area. The latter can be considered an evaluation of centrality [33], [34], highlighting patches and dispersal areas that are essential to the maintenance of connectivity among all patches in a landscape.

In addition to showing which individual pixels are important for network connectivity, we used the cumulative current map to derive two metrics of centrality specific to habitat patches. First, we summed current passing through all pixels within each patch as it traveled between other pairs of patches in the network. This gave an index of the importance of each patch in providing connectivity among all remaining patch pairs, i.e., its importance as a stepping-stone for keeping the network connected. Second, we divided these values by patch area to produce an area-weighted centrality score. This indicated the average importance of pixels within each patch, which can highlight patches that contribute more to network connectedness than would be expected based on their size alone.

## Results

### Habitat Quality and Permeability

Among experts, scores for both habitat quality and permeability assigned to dominant land cover types were reasonably consistent (as indicated by the SD of the scores; Table 1). Experts scored woodland and forest as the most important types, followed by riparian and shrub-dominated cover types. Cover types with the lowest scores were those typically associated with human activity, including developed and agricultural areas. In general, as terrain ruggedness increased, so did its perceived importance as a feature of high habitat quality and permeability. Both canyon bottoms and ridgelines were topographic positions important for defining high habitat quality and permeability. As road density, distance to water, or human population density increased, there was substantial agreement that habitat quality and permeability declined in a linear fashion. Experts also agreed that major highways and large water bodies were extremely poor attributes of habitat and effective barriers to puma dispersal, and that large canals and ditches were somewhat less important barriers.

For defining relatively high habitat quality and permeability, experts agreed that COVTYPE was the most important variable and it received the largest swing weight (0.28 and 0.31 for quality and permeability, respectively; Table 2). The RUGGED variable also was deemed important, with quality and permeability swing weights of 0.23 and 0.22, respectively. WATER was identified as the next most important variable for defining habitat quality (swing weight = 0.16), although TPI was identified as more important for defining permeability (0.18). These results indicate that habitat quality and permeability were not equivalent metrics.

**Table 2:** Average (±1SD) of the expert-defined importance values and swing weights for the habitat variables (i.e., data layers) used to estimate habitat quality and permeability for dispersing pumas in Arizona and New Mexico.

	Quality		Permeability	
Habitat variable^1^	Importance value	Swing weight	Importance value	Swing weight
COVTYPE	950.0 (100.0)	0.277	1000.0 (0.0)	0.305
RUGGED	800.0 (182.6)	0.234	733.3 (208.2)	0.223
BARRIERS	312.5 (131.5)	0.091	133.3 (104.1)	0.041
TPI	475.0 (263.0)	0.139	600.0 (173.2)	0.183
ROADS	250.0 (100.0)	0.073	233.3 (57.7)	0.071
WATER	562.5 (197.4)	0.164	450.0 (218.0)	0.137
HUMANS	75.0 (50.0)	0.022	133.3 (57.7)	0.041

Table 1.^1^ Variable abbreviations defined in

**Figure 2:**
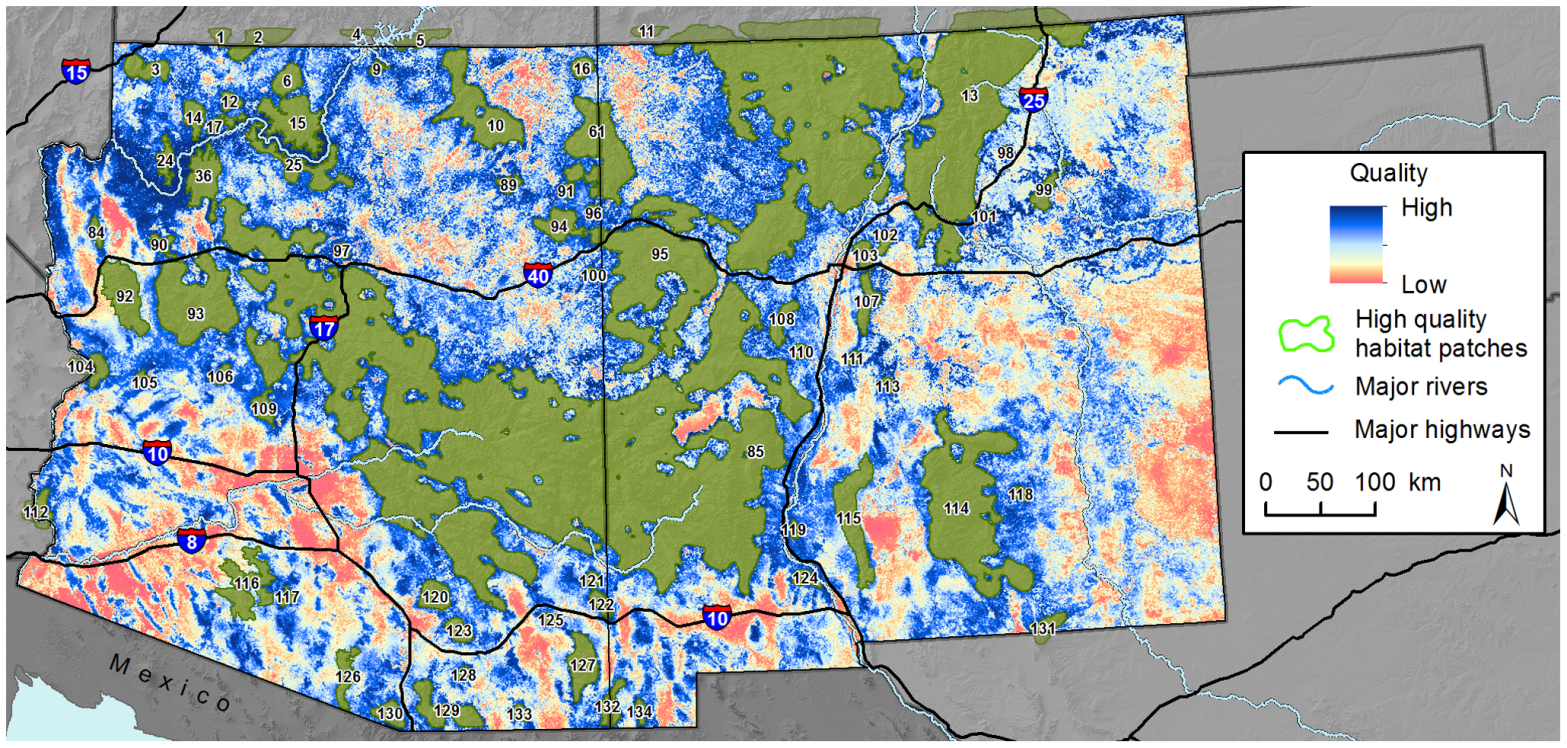
Map of habitat quality and high quality habitat patches for pumas. Estimates of habitat quality were derived using expert-elicited information and seven habitat variables. High quality habitat patches are uniquely numbered for reference. Background maps were produced using data obtained from the U.S. Geological Survey’s Earth Resources Observation and Science Center.

**Figure 3:**
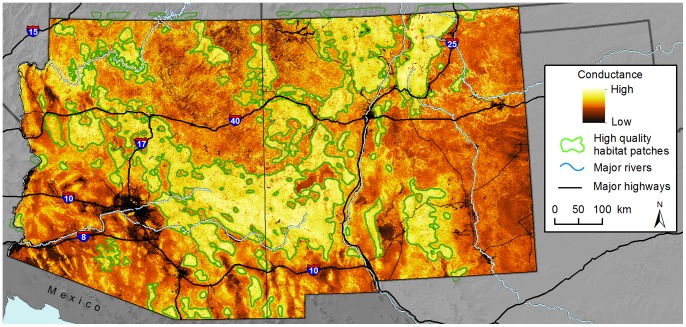
Map of mean habitat conductance for pumas. Estimates of habitat conductance were derived using expert-elicited information and seven habitat variables. High quality habitat patches are uniquely numbered for reference. Background maps were produced using data obtained from the U.S. Geological Survey’s Earth Resources Observation and Science Center.

Cell values for our final habitat quality layer ranged from 153.1–789.3 (mean = 514.9, SD = 112.5; Fig. 2). Cell values for the final habitat permeability (or conductance) layer ranged from 17.5–865.1 (mean = 626.7, SD = 132.5; Fig. 3). Using the habitat quality layer, we identified 67 high quality patches across the study area that ranged from 100.4–68,365.6 km^2^ (total area = 161,855.0 km^2^, mean = 2,415.8, SD = 9,450.9). To minimize computation time, we did not include habitat patches <100 km^2^. The largest contiguous patch (#85 in Fig. 2) occurred in the center of the study area and included large expanses of forest and woodland habitats in the more rugged portions of the Mogollon Plateau. This patch was also contiguous with Arizona’s Galiuro and Winchester Mountains to the south, and the San Francisco Mountains and Black Range to the east into southwestern New Mexico. Many other prominent mountain ranges were encompassed by large habitat patches, including the Hualalapai (92) and Aquarius (93) Mountains in northern Arizona, Santa Catalina (120) and Chiricahua (127) Mountains in southern Arizona, Sacramento Mountains (114) in south-central New Mexico, and portions of the Sangre de Cristo mountain complex in northern New Mexico (13).

### Habitat Connectivity

**Figure 4:**
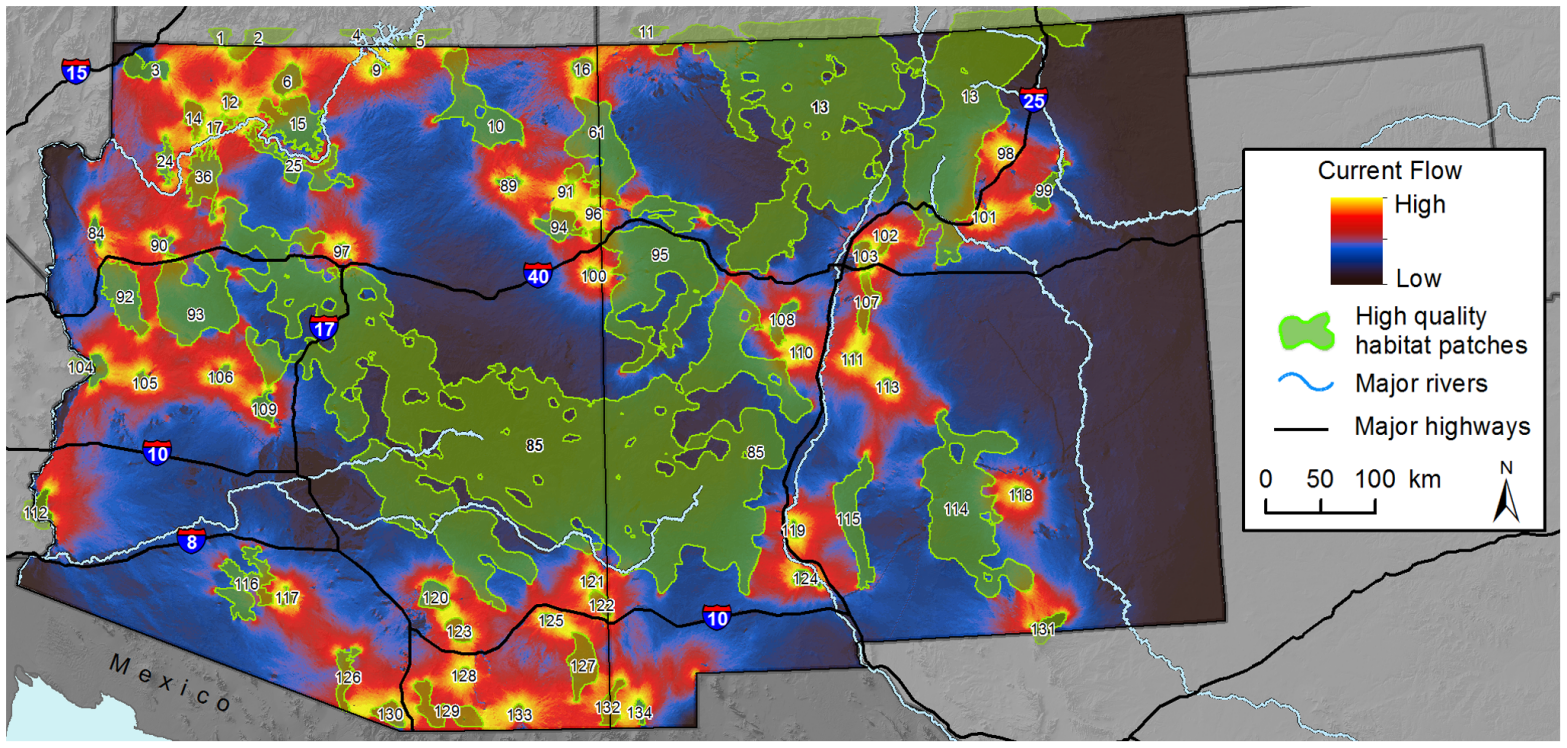
Model of maximum current flow used to estimate connectivity for pumas. Maps were displayed using a histogram equalize stretch. High quality habitat patches are uniquely numbered for reference. Background maps were produced using data obtained from the U.S. Geological Survey’s Earth Resources Observation and Science Center.

Our map of maximum current flow between patches highlighted possible pinch points for connectivity for animals moving directly between patch pairs (Fig. 4). We observed pinch points along the Interstate 40 transportation corridor (from west to east) between patches 84 and 92, 90 and 93, 85 and 97, and 103 and 107. Similarly, along the Interstate 25 or Rio Grande corridor, pinch points may be present (from north to south) between patches 110 and 111, 119 and 85, and 119 and 124.

**Figure 5:**
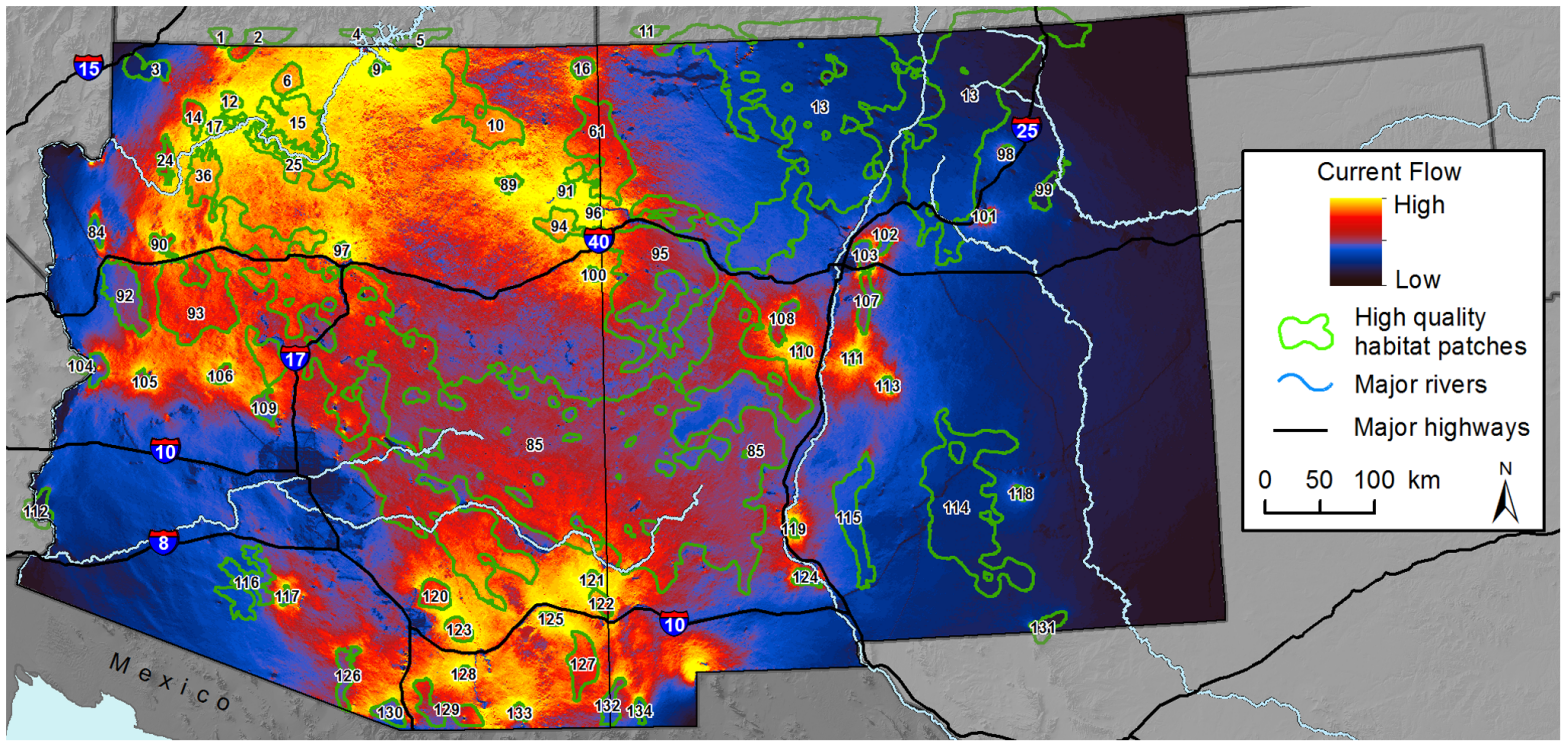
Model of cumulative current flow used to estimate connectivity for pumas across the entire network of habitat patches. Maps were displayed using a histogram equalize stretch. Background maps were produced using data obtained from the U.S. Geological Survey’s Earth Resources Observation and Science Center.

Our map of cumulative current flow (Fig. 5) shows the sum of currents when all patch pairs are iteratively connected, and highlights areas with high centrality, i.e. areas important for keeping the entire network of patches connected. Cumulative current flow was highest in Arizona north of the Colorado River and around Grand Canyon National Park, and in the southeastern corner of Arizona in the Sky Islands region (Fig. 5, Table S3). In Arizona, current flow also was relatively high in west-central areas of the state (see Fig. 1) and across the northeastern corner of the state (Navajo Nation lands). Compared to Arizona, current flow across New Mexico was relatively low, because fewer discrete habitat patches were mapped there. Patterns of high flow were present along the Arizona-New Mexico state line to the north and south. In both cumulative and maximum current flow maps, we observed a ‘halo’ effect around many small patches reflecting high current flow around their perimeter.

**Figure 6:**
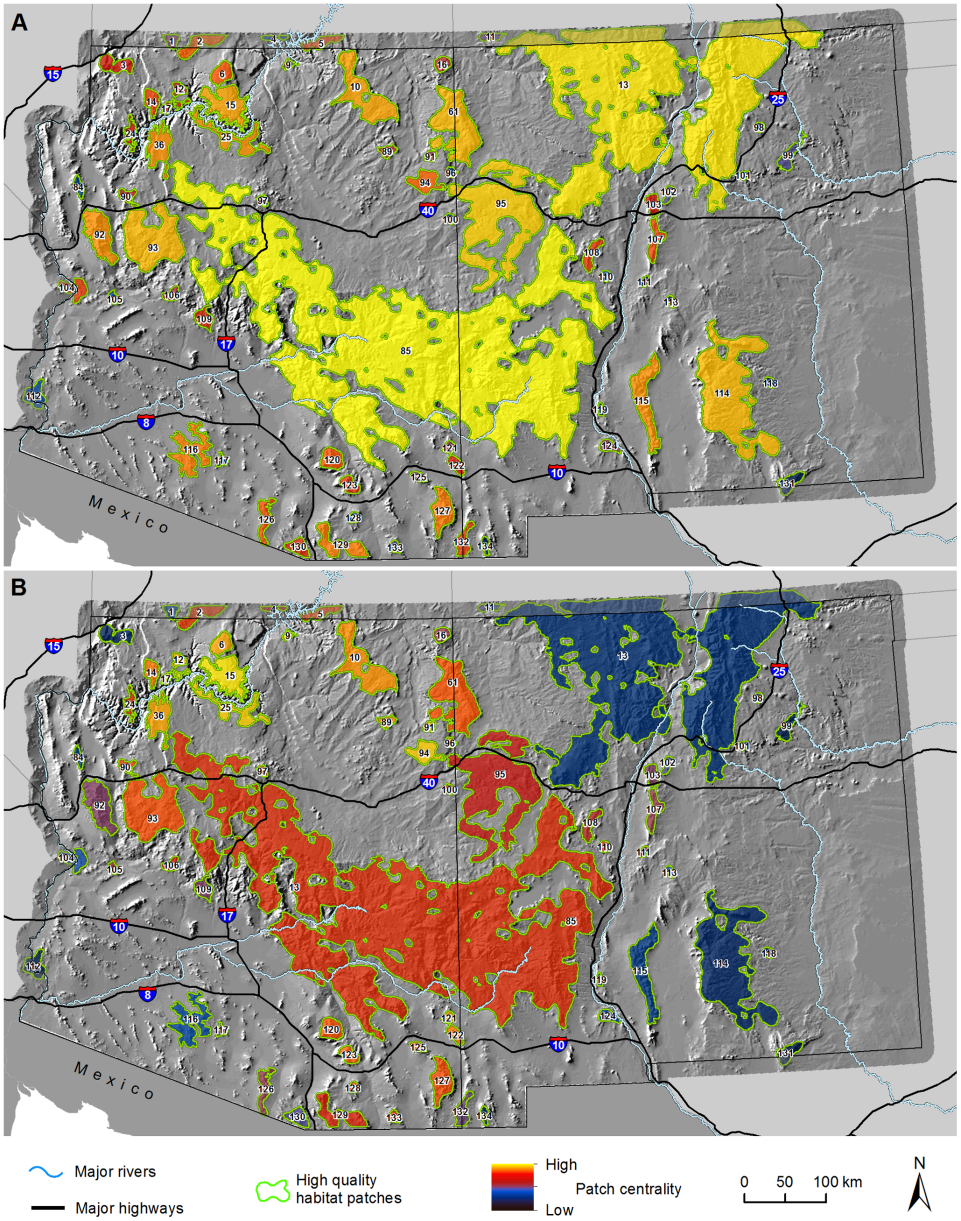
Models of habitat patch centrality. A) Centrality scores derived from summing total current flow across all pixels in each patch as it passes between all other patch pairs. B) Area-weighted centrality scores obtained by dividing scores in panel (A) by patch area. Background maps were produced using data obtained from the U.S. Geological Survey’s Earth Resources Observation and Science Center.

Our maps of habitat patch centrality (Fig. 6) revealed numerous areas that may be important for keeping the overall network of habitat patches connected. Estimates of cumulative current flow highlighted those patches most important for maintaining relatively high current flow between other patches (Fig. 6A). Area-weighted estimates of centrality revealed patches that provided more connectivity value across the network than would be expected by their size alone (Fig. 6B). Multiple large patches in the central portion of the study area (85, 93, 95) and relatively small patches in the northwest (6, 14, 15, 17, 25) and southeast corners of Arizona (e.g., 120, 127) were most important to maintaining overall connectivity, irrespective of the centrality estimate used. Patches in southwestern Arizona (112, 116, 117) and eastern New Mexico (98, 99, 114, 118, 131) were more peripheral and exhibited low centrality, indicating that the removal of any of these particular patches would disrupt overall network connectivity to a lesser degree here than elsewhere. Nevertheless, some patches–particularly in southwestern Arizona and northern Arizona and northern New Mexico–have low centrality scores because they are peripheral to our study area, but likely provide important stepping-stones to habitat patches in Mexico, Utah, and Colorado, respectively.

## Discussion

Our models and maps present a first approximation of habitat quality and connectivity for dispersing pumas in the Southwest region. Several factors contribute to the patterns we observed in our models. For example, model-based estimates of high-quality habitat patches indicated that these areas typically were in relatively remote and rugged terrain and dominated by woodland, forest, chaparral, or riparian land cover types. These outcomes are not surprising since pumas and their prey prefer to occupy topographically complex natural landscapes that are less influenced by humans [9], [15], including areas at the wildland-urban interface [30], [35]. In southern California, telemetered pumas used a similar set of habitats, preferring woodlands, scrublands and riparian areas over grassland or more human-disturbed areas, and were found to use higher elevations more often than lower elevations [26], supporting our expert-based model of how pumas may disperse through landscapes of the Southwest.

Maps of current flow between neighboring patch pairs using the method of maximum pairs are useful for identifying important portions of linkage zones between particular sets of habitat patches, removing potentially confounding effects of network configuration. For example, there were important pinch points along the Rio Grande, where local habitat features facilitate east-west movement.

The cumulative current flow model simultaneously highlights constrained portions of linkage zones and areas important for maintaining a connected network of habitat patches across the entire study region (Fig. 5). For example, current flow between the large habitat patch that includes the Mogollon Rim (patch 85) and the Sacramento Mountains (114) (Fig. 4) illustrates how intermediate habitat patches facilitate connectivity between these two distant habitat patches. Here, current flow was broadly distributed, owing in part to the large surface area of these two habitat patches, and thus the presence of multiple potential movement routes between them. However, in particular, the smaller patches encompassing the Oscura (113), San Andres (115), Caballo (119), and Sierra de Las Uvas Mountain regions (124) appear to provide important stepping-stone habitat between the larger Mogollon Rim and Sacramento Mountains patches. The large distance separating the Mogollon Rim and Sacramento Mountains suggests that the San Andres and its smaller neighboring patches are important ‘stepping-stones’, either for individual dispersing pumas or for inter-generational gene flow between the two larger habitat patches [10].

The ‘halo’ effect of high current flow around some small patches that we observed is a common artifact in circuit modeling. The small surface area of these patches results in higher modeled movement probabilities because there are fewer areas to enter and exit the patches. Although maintaining access to patches is important, these areas could be identified by inspection, and should not be mistaken for pinch points, which are much more difficult to identify without modeling.

### Model Interpretation and Observations of Puma Dispersal

Our above assessment of how pumas are predicted to use the region between the Mogollon Rim and the Sacramento Mountains can be partially evaluated using observations from a previous study that documented dispersal events of pumas inhabiting the San Andres Mountains (115) of southern New Mexico. Independent pumas (*n* = 40) born in the San Andres Mountains rarely dispersed distances >200 km and the majority dispersed in a north-south direction along the spine of the mountain range settling within high-quality habitat [10]. Twenty-one females settled only 1.4 home range diameters from their natal range. However, several pumas (*n = *11) dispersed from the San Andres Mountains to points east and west. Five individuals traveled west and two of these crossed both the Rio Grande and Highway 25 to settle in the large central patch (85) encompassing the Mogollon Rim. The region to the west of the San Andres Mountains has relatively high current flow, with suitable patches (119 and 124) that could act as stepping-stones to the much larger and contiguous Mogollon Rim. Indeed, some pumas actually dispersed to and settled in these smaller habitat patches [10]. But six pumas traveled east across the Tularosa Basin, a desert basin of relatively low predicted conductance, to settle within both the Sacramento (114) and Guadalupe Mountains (131). During these dispersal events, pumas moved in a straight line and crossed 45–65 km of desert in <7 days [10].

These observations point to both the strengths and weaknesses of our modeling approach for conservation planning. Our model shows the importance of stepping-stone habitats for pumas moving westward from the San Andres Mountains. However, our maps show relatively low levels of movement through any particular portion of the Tularosa basin. This should not be taken to imply that this area isn’t important for movement. On the contrary, the large surface area of the San Andres and Sacramento Mountains and relatively uniform conductance values between them mean that current, and thus predicted movement probabilities, are spread out across this basin. Interestingly, large mountain ranges within the southern portions of both states are very visible across expanses of desert. In the case of the San Andres-Sacramento linkage, pumas would be able to see one mountain range from the other, and may simply be taking the shortest route. Long-distance perception is not accounted for in models like ours, but is an important factor in dispersal [36]. Nonetheless, our models predict substantial movement across the basin, just that this movement would be spread out rather than concentrated in small areas. Our models also predict important movement routes from the San Andres Mountains to the north and then east and south, which would maximize use of high-quality habitat by allowing pumas to move along the axes of various mountain ranges.

Detailed assessments of puma dispersal patterns are needed to more fully understand the breadth of habitats that pumas may use during dispersal. Understanding the motivation behind such events will further strengthen the use of connectivity maps, such as ours, as tools for conservation planning [37]. For instance, dispersing pumas may travel for brief periods in different directions, killing prey and establishing temporary home ranges (THR) before they finally settle. The ultimate location that they settle relative to where they were born is usually not as simple as the straight-line distance between two points. For example, a female puma dispersed from the Oquirrh Mountains, Utah to the White River Plateau in Colorado, a straight line distance of 357 km, one of the longest recorded dispersal distances for a female puma [38]. However, this female actually travelled over 1,300 km during her dispersal, having crossed one interstate highway and several other federal and state highways, and successfully negotiated four different rivers and established at least three different THRs [38].

### Network Centrality and Large-scale Disturbance

Areas of high cumulative flow indicate zones with high centrality, i.e., areas important for connecting many pairs of high-quality habitat patches, the loss of which can result in multiple habitat patches in the network becoming disconnected ([34]; e.g., the Sky Islands in southeastern Arizona, or the complex of patches in northwestern Arizona). Our maps summarizing centrality values for habitat patches (Fig. 6) further highlight the role each habitat patch plays in keeping the network connected. Patches with high area-corrected centrality scores (Fig. 6B) could be good conservation investments because they play a stronger role in network connectivity than would be expected by their size alone, and because their small area means they may be particularly vulnerable to loss. Recall that all habitat patch pairs were modeled using 1 Amp of current between them, so that large and small patches were treated as equally important. This modeling choice emphasizes keeping small patches connected to the network, but could be altered by allowing more current to flow from larger (presumably more important) habitat patches. Regardless of how patches are weighted, these analyses of network centrality can yield important insights into how the loss of high-quality habitat patches can affect overall network connectedness.

Our models can help identify high-quality patches and forecast impacts to connectivity when such patches are lost owing to catastrophic events. For example, large-scale disturbances, including wildfire and insect outbreak, have increased in magnitude and severity across the western U.S. [39], [40] and could impact or eliminate key habitat areas. Moreover, increasing drought is expected to spur an increase in fire activity and insect outbreaks that will negatively impact the quality and distribution of forests and woodlands [41]. In the Southwest, exceptionally large wildfires, such as the 2011 Wallow Fire (217,741 ha) in eastern Arizona and the 2012 Whitewater-Baldy Complex Fire (120,534 ha) in western New Mexico, negatively impacted extensive areas of high-quality habitat for pumas, their prey, and other species of conservation concern (e.g., [42]). Each of these fires was the largest in state history and burned extensive areas of the Mogollon Plateau (85). Also in 2011, the Horseshoe Two Fire (90,226 ha) burned large portions of the Chiricahua Mountains (127) in southeastern Arizona. This area comprises the largest high-quality habitat patch in the ecologically important Sky Islands region, which connects genetically distinct subpopulations of pumas [14]. Such changes in forest and woodland structure and distribution will most likely impede landscape connectivity and conservation efforts seeking to sustain habitat for pumas and other species over extensive areas [43].

### Puma Metapopulation Structure in the Southwest

An analysis of the genetic structure of pumas within Arizona, Colorado, New Mexico, and Utah revealed complex metapopulation structure, with a relatively clear genetic discontinuity occurring between regions that constitute the southern Rocky Mountains versus regions further south characterized as the Sky Islands [14]. Within the Sky Islands region, more complex genetic structure was evident with recognized subpopulations in southeastern Arizona that were distinct from those in southern New Mexico. In addition, populations of pumas in central portions of Arizona and New Mexico represented a transition between southern populations and those in northern Arizona and New Mexico, which were genetically more similar to subpopulations in Colorado and Utah. These latter subpopulations were part of the larger core population inhabiting the southern Rocky Mountains. Our habitat models, in general, supported this hierarchical level of genetic structure with low genetic differentiation among sample locations within single habitat patches predicted by our models relative to sample locations between habitat patches [14].

Sustaining puma populations in the Southwest will depend, in part, on the maintenance of the current metapopulation structure, which is a consequence of a network of proximate high-quality habitat patches that support dispersal and gene flow. Some of these patches are very large and contiguous, whereas other patches are small and incapable of supporting viable populations [10], [14]. Although pumas are capable of dispersing over great distances (>1,000 km; [38], [44]), these events are rare and may be symptomatic of patterns of ongoing habitat change or resource management in the West. Protecting or increasing habitat connectedness will be key to maintaining a viable metapopulation in the changing landscapes characterizing the Southwest region [9], [14].

## Conclusions

Successful puma conservation will hinge on land management practices that conserve the integrity of large habitat patches, but also protect the small, high-quality habitat patches that can sustain small puma populations or act as stepping stones which facilitate dispersal. Our identification of high-quality habitat patches and estimates of habitat connectivity can be used to generate hypotheses about the connective value of different habitat components, identify critical pinch points or linkages for animal movement, target the collection of independent data, and inform regional conservation planning efforts at spatial scales that are relevant to key ecological processes such as dispersal [37], [45]. Indeed, these processes will be facilitated by conservation, management, or restoration activities occurring at scales that are congruent with the life history and habitat requisites of wide-ranging wildlife species, including pumas. Furthermore, unlike most other techniques for characterizing landscape connectivity, our approach permits the quantitative and simultaneous evaluation of multiple alternative linkages, which can be used to develop more comprehensive conservation planning strategies. Since our results reflect expert-defined inputs derived at a coarse spatial resolution and do not capture many of the landscape features (e.g., cliff areas) that might preclude localized movements, project-level planning efforts that draw on our results should do so with caution. Approaches that include more in-depth assessments of dispersal and associated fine-scale patterns of habitat use by pumas, as well as more detailed analyses linking genetic relatedness among subpopulations with measures of connectivity, are needed to validate and refine landscape-scale models of habitat quality and connectivity in order for such models to be confidently used to plan for conservation efforts in the region.

## Supporting Information

Figure S1 **Methodological diagram and steps taken to parameterize expert-based models.**
(JPG)

Table S1 **Form used to elicit information on habitat attributes and rankings.**
(DOCX)

Table S2 **Form used to elicit information on habitat variable importance scores and to calculate weights.**
(DOCX)

Table S3 **Matrix of effective resistance values for all possible pairs of 67 high quality habitat patches used to estimate connectivity.**
(XLSX)

Text S1 **Descriptions and qualifications of experts, and instructions provided to elicit information on puma habitat quality and connectivity in the Southwest.**
(DOCX)
