# Correction: Models of Regional Habitat Quality and Connectivity for Pumas (*Puma concolor*) in the Southwestern United States

**DOI:** 10.1371/annotation/3e4cab4a-23b1-4912-b943-9119cb2401aa

**Published:** 2014-01-02

**Authors:** Brett G. Dickson, Gary W. Roemer, Brad H. McRae, Jill M. Rundall

The online version of this article contains the incorrect version of Table 2. Please see the correct Table 2 here: http://plosone.org/corrections/pone.0081898.t002.cn.tif

