# Supplementary figures and images for: Models of Regional Habitat Quality and Connectivity for Pumas (Puma concolor) in the Southwestern United States
Source: PLoS One. 2013 Dec 18;8(12):e81898. doi: 10.1371/journal.pone.0081898 (PMC3867332; doi:10.1371/journal.pone.0081898)

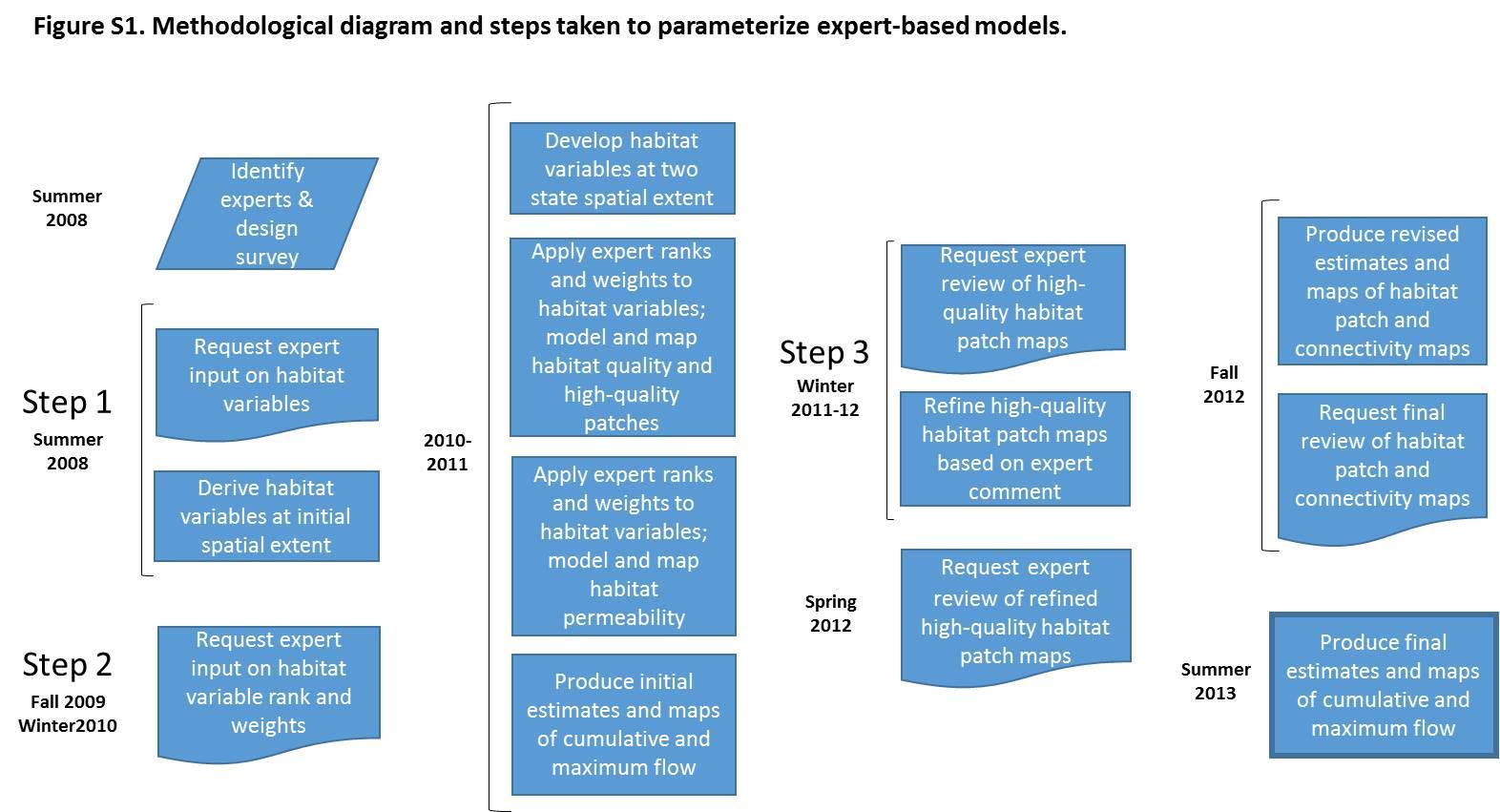

Supplement: Figure S1 — Methodological diagram and steps taken to parameterize expert-based models. (JPG) [file pone.0081898.s001.jpg]
