# Supplementary material for: Models of Regional Habitat Quality and Connectivity for Pumas (Puma concolor) in the Southwestern United States
Source: PLoS One. 2013 Dec 18;8(12):e81898. doi: 10.1371/journal.pone.0081898 (PMC3867332; doi:10.1371/journal.pone.0081898)
Supplement: Table S1 — Form used to elicit information on habitat attributes and rankings. (DOCX) [file pone.0081898.s002.docx]

**Table S1. Form used to elicit information on habitat attributes and rankings. The original spreadsheet and supporting information (Text S1) were provided by email and/or standard U.S. mail. Note that, early in the elicitation process, ‘habitat quality’ was communicated as ‘habitat suitability’ and ‘importance score’ as ‘attribute rank.’**
