# Supplementary material for: Models of Regional Habitat Quality and Connectivity for Pumas (Puma concolor) in the Southwestern United States
Source: PLoS One. 2013 Dec 18;8(12):e81898. doi: 10.1371/journal.pone.0081898 (PMC3867332; doi:10.1371/journal.pone.0081898)
Supplement: Table S2 — Form used to elicit information on habitat variable importance scores and to calculate weights. (DOCX) [file pone.0081898.s003.docx]

**Table S2. Form used to elicit information on habitat variable importance scores and to calculate weights. Totals and weights were calculated automatically using embedded formulas within spreadsheet. The original spreadsheet and supporting information (Text S1) were provided by email and/or standard U.S. mail. Note that, early in the elicitation process, ‘habitat quality’ was communicated as ‘habitat suitability.’**
