# Supplementary material for: Models of Regional Habitat Quality and Connectivity for Pumas (Puma concolor) in the Southwestern United States
Source: PLoS One. 2013 Dec 18;8(12):e81898. doi: 10.1371/journal.pone.0081898 (PMC3867332; doi:10.1371/journal.pone.0081898)
Supplement: Text S1 — Descriptions and qualifications of experts, and instructions provided to elicit information on puma habitat quality and connectivity in the Southwest. (DOCX) [file pone.0081898.s005.docx]

**Text S1. Descriptions and qualifications of experts, and instructions provided to elicit information on puma habitat quality and connectivity in the Southwest.**

**Descriptions and qualifications of experts**

*Jack Childs*

Jack studied the habits and preferred habitat of pumas while training hounds and hunting the species in Arizona and New Mexico from 1963 to 2007. From 1997 until 2009, he was a member of the Arizona/New Mexico Jaguar Conservation Team where he served as chairman of the depredation committee, chairman of the research committee, and was the spokesperson at community forums sponsored by the outreach committee. Jack is now serving as the field coordinator for a 3 year camera trapping study of the habitat, the prey, and the competitors of the jaguar along the border of Arizona/New Mexico and Sonora, Mexico. He recently authored a book, *Tracking the Felids of the Borderlands,* which details the differences in the depredation habits and sign of jaguar and pumas, and has co-authored several research papers on jaguars and pumas.

*Warner Glenn*

Warner is a lifelong resident of the Malpai Borderlands region and a founding member of the Malpai Borderlands Group. With his wife, Wendy, he owns and manages two ranches: the Malpai Ranch on the Mexican border and his family's historic J-A Ranch in the Chiricahua Mountains. Along with his wife and daughter, he also runs a private hunting guide enterprise. Warner is the author of *Eyes of Fire: Encounter with a Borderlands Jaguar*. He has been hunting pumas and other fauna of the region for 65 years.

*Ken Logan*

Ken is a Wildlife Researcher with Colorado Parks and Wildlife. He earned his Ph.D. in Wildlife Sciences at the University of Idaho in 2001. He has been involved in puma research, including population ecology, behavior, puma-prey relationships, social organization and puma-human interactions since 1981. Ken studied puma population dynamics, behavior, and social organization for 10 years in New Mexico and is currently (2013) in this second 10-year research program on puma population ecology in southwestern Colorado. He has co-authored several related professional papers and books including *Desert Puma: Evolutionary Ecology and Conservation of an Enduring Carnivore* (2001).

*Harley Shaw*

Harley grew up in Arizona and started with the Arizona Game and Fish Department in 1955. He completed a BS degree in wildlife management at the University of Arizona and a MS degree in Wildlife Management at the University of Idaho. From 1963 to 1990, he worked as a research biologist for the Arizona Game and Fish Department, carrying out projects on mountain lions and other species. His earlier work on lions led to publication of A Mountain Lion Field Guide. Harley has authored and co-authored numerous works on mountain lions in peer-reviewed journals and popular articles. One of the most popular being the book, *Soul Among Lions*. He also was one of 13 members of a working group that wrote and published the Cougar Management Guidelines in 2005. Harley is the current Editor-in-Chief of the Wild Felid Monitor.

*Linda Sweanor*

Linda earned her M.S. in Wildlife Sciences at the University of Idaho in 1990. She has been involved in puma research, including population ecology, puma-prey relationships, puma social organization and puma-human interactions, since 1985. Linda has studied pumas in New Mexico and southern California. She has co-authored several related professional papers and also co-authored *Desert Puma: Evolutionary Ecology and Conservation of an Enduring Carnivore* (2001). More recently, Linda assisted on felid (puma, bobcat) research projects for Colorado State University and Colorado Parks and Wildlife. Linda is a founding member and current President of the Wild Felid Research and Management Association.

**Contact sequence, methodological steps, and instructions provided to experts in order to elicit information on puma habitat quality and connectivity. All instructions and maps were provided by email and/or standard U.S. mail. The below paragraphs were excerpted from these communications. Note that, early in the elicitation process, ‘habitat quality’ was communicated as ‘habitat suitability,’ ‘importance score’ as ‘attribute rank,’ and ‘patches’ as ‘cores.’**

*Contact 1: Identify habitat variables (Step 1)*

For this first phase, we are only requesting that you to identify up to four landscape-scale variables that are likely to influence puma habitat suitability and permeability. Of course, we are asking that you make some generalizations about puma habitat, but feel free to recommend these variables based only on the knowledge you have and the places you know. For example, dominant vegetation type, topographic position, and distance from major highways are variables that are often used to characterize these factors. You are also free to recommend the same or different variables for each of these factors, as well as any specific classes within each variable (e.g., forest, woodland, grassland vegetation; high slopes, low slopes, canyons). During the second phase of this work, and once we have compiled the GIS variables that reflect those you have selected, we will task you with ranking and weighting these individual classes.

*Contact 2: Variable and attribute ranking and weighting (Step 2)*

Habitat variable attribute ranking – please rank (between 0 and 1000) the individual attributes for each habitat variable separately for both habitat suitability and permeability. You will complete this in the ‘attribute ranking’ worksheet of the provided spreadsheet. Essential to our modeling approach is a ranking of the relative importance of classes, or “attributes” within 3 of the 7 primary habitat variables: 1) vegetation communities, 2) topographic position, and 3) barriers. Please note that we are not asking you to rank the classes within the four identified habitat variables of terrain ruggedness, water feature density, road density or human population density, instead favoring to use 10 quantile classes (i.e., 100, 200,…, 1000). See the attached spreadsheet for the full list of attributes for each variable.

Weighted order of importance – please weight (between 0 and 1000) the relative importance of each of seven habitat variables in contributing separately to both suitability and permeability. You will complete this in the ‘habitat weighting’ worksheet of the provided spreadsheet. Please assign a weight of importance to the seven individual habitat variables identified above: 1) vegetation communities, 2) topographic position, 3) barriers, 4) terrain ruggedness, 5) water feature density, 6) road density, and 7) human population density. Again, we rescaled and ranked these latter four variables using 10 quantiles: 100, 200, 300,…, 1000.

*Contact 3: Expert review and comment on high quality habitat patches or ‘cores’ (Step 3)*

Step 3: The attached core area map has eighty-six cores, or habitat ‘stepping stones,’ ranging from 17 to 60,652-hectares in size. This model was developed by applying the habitat information you provided in 2008-09, using updated spatial data layers, across this new study area. Based on your knowledge of puma in the Southwest, we are looking for specific input on which cores might be removed or modified, as well as indications of where there may be missing cores on this landscape. (Digital and hard copy maps provided for review)

*Contact 4: Expert review of integrated comments of high quality habitat patches; delivery of map-based estimates of both connectivity models (Step 3)*

We have integrated your comments on the ‘high quality’ habitat patch map into the new version of the connectivity models for Arizona and New Mexico. We have attached both the circuit-theoretic models of maximum pairwise and network centrality and landscape connectivity for puma across Arizona and New Mexico for your review. Beyond these bi-state models, we are asking you to select sets of cores to test in pairwise comparison, i.e., more local case study areas.* These pairs or clusters (<5) of patches, and their corresponding local connectivity models, will be the basis for more focused inference. We ask that you draw on the patches that you have just refined in this current map-based step, and that you believe would be of high interest for taking a closer look puma pathways in a more familiar conservation context (please identify them by number). We have selected focal areas for your review (if you have a different focal area in mind, let us know).

*Contact 5: Expert review and comments on refined high quality habitat areas and identify pairs for local analysis (Step 3)*

For this effort we are asking for two areas of feedback, one is to review the new round of enumerated high quality habitat patches depicted on the enclosed map, the second is to select pairs or clusters (<5) of patches, that we can test in a pairwise comparison, i.e. more local case study areas. Beyond these bi-state models, we are asking you to select sets of cores to test in pairwise comparison, i.e., more local case study areas.* These pairs or clusters (<5) of patches, and their corresponding local connectivity models, will be the basis for more focused inference. We ask that you select from the patches that you have just refined in this current map-based step, and that you believe would be of high interest for taking a closer look at puma pathways in a more familiar conservation context.

*These comparisons were not conducted as part of the work submitted for peer review.
